# Editorial for Special Issue: Nanostructured Surfaces and Thin Films Synthesis by Physical Vapor Deposition

**DOI:** 10.3390/nano11010148

**Published:** 2021-01-09

**Authors:** Alberto Palmero, German Alcala, Rafael Alvarez

**Affiliations:** 1 Materials Science Institute of Seville (CSIC-US), Américo Vespucio 49, 41092 Seville, Spain; alberto.palmero@csic.es; 2 Departamento Ingeniería Química y Materiales, Universidad Complutense de Madrid, Avenida Complutense s/n, Facultad de Ciencias Químicas, 28040 Madrid, Spain; galcalap@ucm.es; 3 Departamento de Física Aplicada I. Escuela Politécnica Superior, Universidad de Sevilla, c/Virgen de África 7, 41011 Seville, Spain

The scientific interest in the growth of nanostructured surfaces and thin films by means of physical vapor deposition (PVD) techniques has undoubtedly increased in the last decade [1]. Even though some of them can be considered mature, as they were first implemented and analyzed a few decades ago [2], the progressive understanding of fundamental atomistic phenomena [3,4], as well as their popularity in the technological industry, have prompted the scientific community to propose novel growth methodologies/geometries which, based on the classic approaches, have bestowed an even larger versatility in terms of nanostructural possibilities. In this way, film nanostructures that were unthinkable years ago are now possible by PVD: for instance, a classical technique such as the magnetron sputtering (MS) deposition method, that originally aimed to produce highly compact coatings, is currently being explored for the production of highly porous thin films thanks to the oblique angle deposition geometry [5,6]. Moreover, an MS-based methodology named High Power Impulse Magnetron Sputtering (HiPIMS) is employing high power pulsed electromagnetic signals to produce highly compact films, expanding the classic methodology [7]. Hence, it can be said that the research and application of PVD techniques are living a scientific golden age due to the large amount of exciting new possibilities, fundamental discoveries, and the numerous potential applications based on their wide range of morphological characteristics and properties, e.g. in photovoltaic cells [8], tribological coatings [9,10], optofluidic sensors [11], energy storage [12], etc. 

Unfortunately, the processes responsible for the formation of a certain nanostructure by PVD are, in most of the cases, not yet known with the sufficient depth to gain enough level of control to optimize its functionality when incorporated into devices. It is then necessary to study novel nanostructures and their properties as well as the nanostructuration mechanisms, by addressing fundamental issues to understand them. In this special number, ten research works analyze various relevant aspects concerning new approaches and methodologies based on classic PVD techniques to produce films with singular nanostructures and morphologies, representing the current state of the art in this research field. Among them, two important review papers are included allowing any potential reader to understand the most relevant advances in the following two different topics. In ref. [13] the growth of metallic ultra-thin films by continuous dynamic monitoring is reviewed in depth, by combining in situ and real-time optical and electrical probes to analyze the first stages of growth in the MS deposition of a large number of metals with different crystalline structures. In ref. [14], a review on the growth of nanostructured surfaces with plasmonic properties, obtained by the combination of nanosphere lithography and PVD, is presented. There, the authors analyze the recent advances linking the fabrication routes, the film nanostructure and its plasmonic properties, with special emphasis on its application for the early detection of hepatocellular carcinoma using surface-enhanced Raman scattering and controlling the growth of Ag nanoparticles. 

In addition to these relevant review manuscripts, two original research works are presented [15,16]. In ref. [15] the authors study nanostructured thin films grown by MS deposition at oblique angles, in this case using tungsten. These films are characterized by a porous structure consisting of tilted nanocolumns. By varying the deposition conditions, the tilt angle, thickness and separation of these columns can be affected, which in turn affects the optical, electrical or mechanical properties of the film. Here, the authors study the effect of both the film thickness and the gas pressure within the deposition chamber on properties such as the electrical resistivity of the film, or the anisotropy of the propagation of 2-dimensional elastic waves along the film. They have found that anisotropy rises at low pressures, where the columnar structures are better defined and more tilted, and that it increases with the thickness of the film. Electrical resistivity was found to decrease with the film thickness and to increase with the gas pressure, due to changes in the crystallinity of the film. A thorough characterization of AZO (Al-doped zinc oxide) thin films deposited by radio frequency (RF) MS is covered in ref. [16]. Sheet resistance, thin film thickness, resistivity, hall mobility, carrier concentration, optical transmittance, and band gap energy were determined for these transparent and conducting thin films as a function of the substrate position within the substrate holder with a 3 mm spatial resolution, for varying conditions of pressure, applied power, and substrate-target distance. The results show a great resistivity dependence with the substrate position, varying about 2 orders of magnitude within a distance of 10 mm. Contrarily, the spatial profile of the transmittance is quite homogenous along the surface of the samples. A reduction of the energetic oxygen ions coming from the erosion track of the target is proposed as a way to produce more homogenous films.

Other works included here focus on fundamental analysis, while also targeting a specific application, such as sensors [17,18], solar cells [19], or biomedicine [20]. In ref. [17] the authors synthesize composite TiO_2_-Ag_2_O nanorods to be used as chemoresistive sensors for the detection of trace amounts of NO_2_ gas. TiO_2_ nanorods were first prepared by hydrothermal methods, and then RF-MS of an Ag target in presence of oxygen was used to deposit Ag_2_O on the TiO_2_ nanorods with different degrees of coverage. The gas-sensing performance of the composite nanorods, when the coverage was made in the form of discrete Ag_2_O particles, was found to be superior to that of pristine TiO_2_ nanorods and even other TiO_2_-Ag_2_O sensors previously reported in the literature. Contribution [18] presents a novel technique that improves Ag nanorods substrates used for surface-enhanced Raman scattering (SERS), rising their sensibility by four times and increasing their thermal stability range by more than 100 ºC. The technique consists of the deposition of an ultrathin capping Al_2_O_3_ layer on the top of the Ag nanorods, followed by the deposition of an additional Ag capping layer on top of the previous one. The Al_2_O_3_ layer improves thermal stability, while the last Ag layer boosts the SERS sensitivity. The Ag nanorods and the capping layers were grown using the electron-beam evaporation deposition technique at oblique angles. A detailed study of the growth of nano-sculpted thin films by the MS technique in glancing angle deposition configuration is displayed in ref. [19]. The effects of key deposition parameters, such as the deposition angle, the gas pressure within the reactor, the temperature of the substrate or the way the substrate is rotated during deposition are experimentally studied, and found to be in agreement with numerical simulations of the film growth. Film properties such as the tilt angle of the grown nanocolumns, the film porosity and, in some cases, its composition and crystalline phase, are also characterized. Finally, the authors propose the integration of nano-sculpted TiO_2_ coatings into the photo-anode of dye-sensitized solar cells, concluding that a hybrid system incorporating both nanocolumns and nanoparticles significantly improves their efficiency. In ref. [20] a novel experimental methodology to produce porous thin films by MS on large surfaces is tested as an alternative to typical oblique angle deposition geometries. For this, two-side implant plates with areas up to 15 cm^2^ were coated with Ti nanocolumns using an industrial reactor. While this was already achieved on small surfaces using laboratory reactors, the relevance of this paper resides on the development of a new geometrical approach to achieve the oblique incidence on industrial reactors as well as the homogeneous coating of a large area plate with Ti nanocolumns. Moreover, the authors demonstrate that the functionality of the obtained porous Ti coatings is maintained, exhibiting the same antibacterial properties as those produced at the laboratory. 

Finally, important applications based on these films are developed for thin film transistors [21] or photovoltaic solar cells [22]. In ref. [21] the authors describe a way to improve the performance and stability of indium–gallium–zinc–oxide (IGZO) thin-film transistors (TFTs), which are widely used in active-matrix displays. By pretreating the substrate (a SiO_2_ buffer layer) with F-plasma in a reactive ion etching chamber before depositing a 30 nm IGZO layer by magnetron sputtering, they describe the formation of indium fluoride nanoparticles in the interface, which increases the density of the IGZO, thus improving mobility and bias stability of these oxide TFTs, and allowing for their fabrication at lower temperatures. In order to improve the characteristics and efficiency of a photovoltaic solar cell, in ref. [22] the authors introduce a Cu_2_ZnSnSe_4_ (CZTSe) nano-layer between the metallic (Mo) electrode contact layer and the active absorbing perovskite layer (MAPbI_3_). This nano-layer was deposited by RF-MS and was later subjected to an annealing process, which greatly improved its hole mobility and its role as heterojunction layer.

As derived from the ten works presented in this special number, the PVD technology comprises a family of several deposition techniques, each one of them embracing a number of different geometric configurations and deposition parameters with a direct effect on the nanostructure, morphology and properties of the produced film. It also allows deposition of most metals and many ceramic materials on a wide variety of substrates, since the deposition temperature is low enough to avoid modifications during the process on most substrate material candidates. Additionally, this is a technology with an important presence in several industrial sectors, which confirms that many scalability and production issues are already overcome. All these features provide the possibility of new developments in a huge range of possible applications, some of them already broadening like among others solar cell components, electronic and photonic applications, gas, liquid and pressure sensors and actuators, biosensors, cell-surface interaction, piezoelectric nanogenerators, electrochromic applications, water splitting fuel cells and hydrogen storage, Li-ion batteries, photovoltaic applications, surface controlled wettability, nanocarpet effect, anisotropic wetting, etc.

However, in order to reach a given surface nanostructure and architecture, and its consequent set of desired properties, a deep understanding at an atomistic level of the deposition processes taking place in both the gaseous and the solid phases involved is needed. The works published in this special number represent an excellent illustration of the recent achievements reached by means of the combination of fundamental/applied experimental research and numerical modelling, increasing this knowledge and enabling the progress in new tailored nanostructured surfaces and thin films. The potential that these developments suggest for the future of surface engineering can only be glimpsed nowadays.

## Data Availability

Not applicable.
